# Photodynamic therapy mediated immune therapy of brain tumors

**DOI:** 10.20517/2347-8659.2018.31

**Published:** 2018-07-10

**Authors:** Henry Hirschberg, Kristian Berg, Qian Peng

**Affiliations:** 1 Beckman Laser Institute and Medical Clinic, University of California, Irvine, CA 92617, USA.; 2 Department of Radiation Biology, Norwegian Radium Hospital, Oslo University Hospital, Montebello, Oslo N-0310, Norway.; 3 Department of Pathology, Norwegian Radium Hospital, Oslo University Hospital, Montebello, Oslo N-0310, Norway.

**Keywords:** Photodynamic therapy, photochemical internalization, photodynamic therapy induced cell death, anti-brain cancer vaccine

## Abstract

Photodynamic therapy of tumors requires the topical, systemic or oral administration of a photosensitizing compound, illumination of the tumor area by light of a specific wavelength and the presence of oxygen. Light activation of the photosensitizer transfers energy to molecular oxygen creating singlet oxygen, a highly reactive and toxic species that rapidly reacts with cellular components causing oxidative damage, ultimately leading to cell death. Tumor destruction caused by photodynamic therapy is not only a result of direct tumor cell toxicity via the generation of reactive oxygen species but there is also an immunological and vascular component involved. The immune response to photodynamic therapy has been demonstrated to significantly enhance its efficacy. Depending on a number of factors, including type of photosensitizer, light dose and dose rate, photodynamic therapy has been shown to induce cell death via apoptosis, necrosis, autophagy and in particular immunogenic cell death. It is the purpose of this review to focus mainly on the role photodynamic therapy could play in the generation of specific anti-tumor immunity and vaccines for the treatment of brain tumors.

## INTRODUCTION

Tumor resection is the primary treatment employed in the treatment of high grade gliomas (HGG). The main functions of surgery are decompression of the brain, obtaining a histopathological and molecular classification of the tumor and reducing tumor load, allowing maximum effect of postoperative therapy. Despite employing modern imaging and surgical techniques, that increase the incidence of gross tumor resection, the tumor invariably recurs, usually in the vicinity of the surgical resection cavity^[1,2]^. The therapeutic goal following gross surgical resection of brain tumors therefore is to prevent recurrence by eliminating the infiltrating tumor cells both remaining in the margins of the resection cavity as well as remote from the tumor site. With this goal in mind anti-cancer immunotherapy is being actively researched as an important therapeutic modality for the treatment of HGG. Of the several methods to induce effective anti-tumor immune response, photodynamic therapy (PDT) has some potentially unique properties. PDT can induce combinations of apoptosis, autophagy and necrosis and immunogenic cell death, depending on a number of factors including type of photosensitizer, light dose and dose rate. It is the purpose of this review to focus mainly on the studies related to PDT-generated anti-tumor immunity and vaccines for gliomas.

### Photodynamic therapy

**Figure 1.**
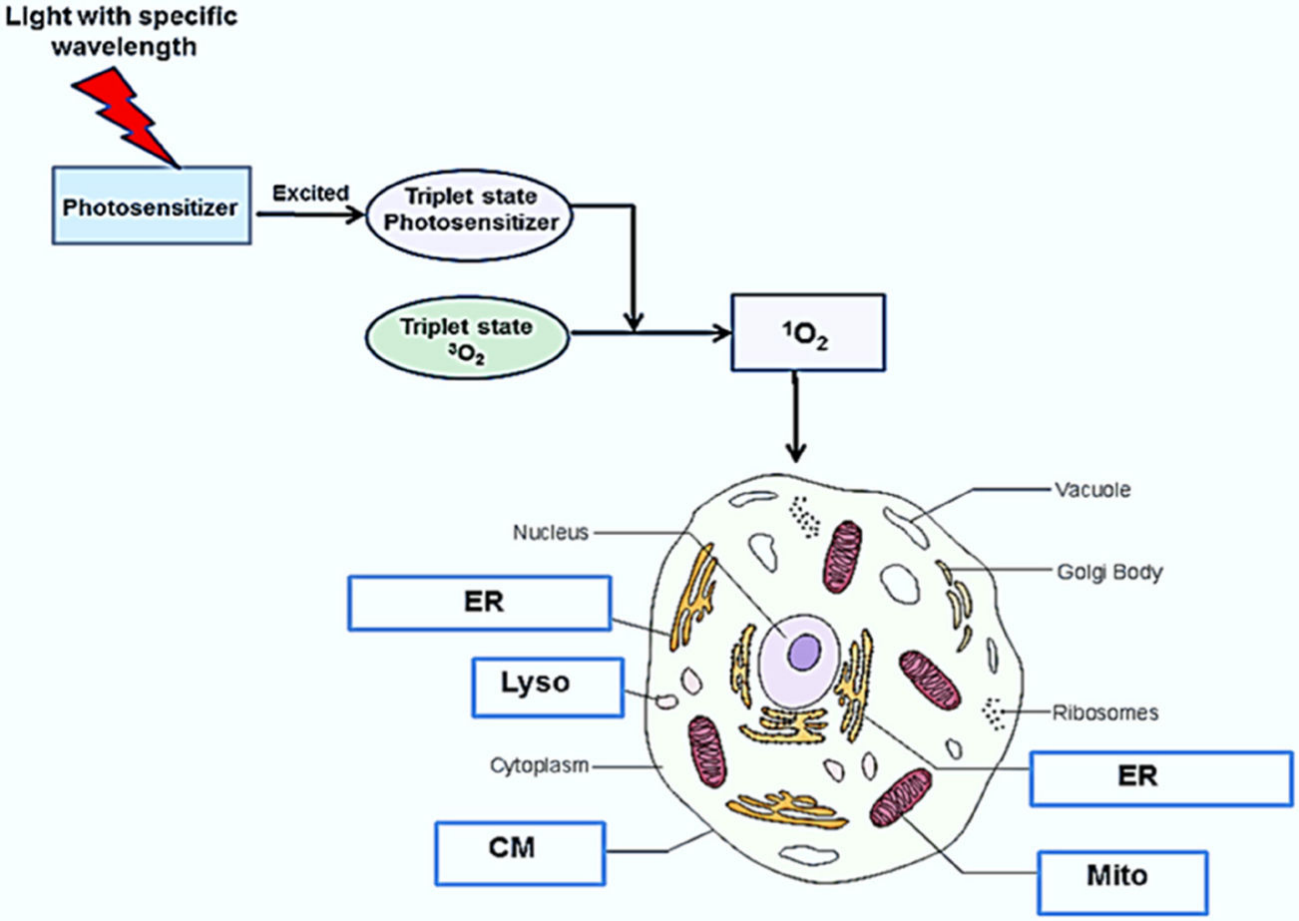
Mechanism and targets for photodynamic therapy. Following photosensitizer administration, light of a particular wavelength matching an absorption resonance of the photosensitizer, is used to excite the molecule up to a triple state. The excited photosensitizer transfers energy to ground state molecular oxygen (^3^O_2_) resulting in the generation of singlet molecular oxygen (^1^O_2_), a potent reactive oxygen species, resulting in cell death. cell membrane (CM) mitochondria (Mito), endosome, lysosome (Lyso), and endoplasmic reticulum (ER)

PDT of tumors requires the topical, systemic or oral administration of a photosensitizing compound, illumination of the tumor area by light of a specific wavelength and the presence of oxygen^[3-8]^. The photon energy activates the photosensitizer and initiates a complex photochemical reaction that generates cytotoxic reactive oxygen species (ROS) as shown in Figure 1. The light activated photosensitizer (PS) transfers energy to molecular oxygen creating singlet oxygen, a highly reactive and toxic species that rapidly reacts with cellular components causing oxidative damage, ultimately leading to cell death. Singlet oxygen causes mainly membrane damage by oxidizing amino acids, unsaturated fatty acids and cholesterol. Both the cell membrane as well as intracellular membranes such as mitochondria, endo-lysosome and endoplasmic reticulum damage is induced by PDT is largly dependent on the type of photosensitizer used^[8]^.

Different photosensitizers react with specific intracellular organelles, resulting in cell death via several varied mechanisms [Table 1].

Unlike ionizing radiation and many chemotherapeutic agents, PDT does not exert its effects via DNA damage^[7]^. Additionally, PDT is a highly site-specific form of treatment, since its effect is restricted to the immediate vicinity of the region of illumination.

## PDT TREATMENT INDUCED ANTI-TUMOR IMMUNITY

Tumor destruction caused by PDT is not only a result of direct tumor cell toxicity via the generation of ROS but it is well established that there is also a significant immunological component involved. The great majorities of experimental studies have been done on extra-cranial tumors, and are reviewed in several extensive recent publications^[9-14]^.

The first evidence for induction of a tumor-specific immune response came by the demonstration that normal mice cured by PDT were able to resist a re-challenge with tumor cells in a tumor-specific manner^[15]^ while immunosuppressed counterparts were not able to resist the re-challenge. Induction of systemic and memory immunity following PDT treatment has been verified in numerous studies. Systemic immunity following PDT treatment has been demonstrated by the ability of a locally induced immune response to affect distant non-treated areas^[16-19]^. Treatment of subcutaneous (s.c) primary tumors that led to 90%-100% of tumor ablation after PDT treatment showed a significant reduction of metastasized lung tumors compared to non-treated controls. In particular, a study using s.c colon carcinoma treated with hypericin-PDT yielded 100% of tumor cures, and i.v. re challenge with viable tumor cells showed no development of new tumors^[20]^.

PDT has been shown to induce apoptosis, necrosis, autophagy and immunogenic cell death (ICD)^[21]^. ICD is a cell death mode where the dead and dying cancer cells expose and/or release damage associated molecular patterns (DAMPs). Although DAMPs are present in cells under normal conditions, they are exposed on the cell surface or released from cells upon the damage caused by the ROS generated by PDT. DAMPs reported to be necessary for the generation of antitumor immunity and induced upon PDT include surface calreticulin (CRT), heat shock protein (HSP) 70, HSP90, secreted adenosine triphosphate (ATP), and high-mobility group box 1 protein (HMGB1)^[21-25]^. Importantly, DAMPs cause maturation, activation and antigen processing/presentation of APCs, leading to their migration and proliferation in local lymph nodes. The mature APCs in the lymph nodes present the tumor antigens to a specific subset of CDs^+^ T cells.

In addition, several studies have shown that PDT-treatment of extra cranial tumors followed by direct intra-tumoral injection of immature DCs, leads to an enhanced anti-tumor immune response compared to PDT treatment as single therapy^[26-28]^. This strategy induces *in situ* DC activation which enhances antigen acquisition and processing as well as migration of the DCs to draining lymph nodes.

### Photochemical internalization

Photochemical internalization (PCI), a derivative of PDT, has been shown to increase the efficacy of drugs, gene transfection as well as a variety of other anti-cancer agents that are taken up into cells by endocytosis^[29-33]^. PCI is based on the use of specially designed photosensitizers, such as AlPcS2a, TPPS2a, TPcS2a that localize preferentially in the membranes of endocytic and lysosomal intracellular vesicles. Upon exposure to light of appropriate wave lengths, the photosensitizers induce the formation of short range singlet molecular oxygen, destroying the intracellular vesicles membranes, thus leading to the release of the contents of these vesicles into the cell cytosol. The released macromolecules can now exert their full biological activity instead of being degraded by lysosomal hydrolases.

Norum *et al*.^[34]^ (2017) has examined the efficacy of PCI delivery of bleomycin (BLM-PCI) and its impact on systemic anti-tumor immunity in an extra-cranial mouse model. Their results showed that both PDT and BLM-PCI were incapable of inducing a curative effect in athymic mice at the light dose tested. In contrast, 50% of the light dose of that used in athymic mice resulted in a curative effect in 90% of the animals after BLM-PCI and 70% after PDT in normal mice. Inhibition of tumor cell growth was observed when combined with co-injection of splenic T cells from mice treated and cured with BLM-PCI. The anti-tumor immunity induced by BLM-PCI was equal to that obtained with PDT treatment, but at a lower light dose. Furthermore, the induced immune response after BLM-PCI was sufficient to reject tumor re-challenge immediately after PCI and lasted for at least two months.

An additional and novel method for enhancing the efficacy of peptide vaccines in extra cranial studies has been explored by Haug *et al*. ^[35]^ (2018) utilizing PCI to promote the escape of trapped endocytosed peptides into the cytosol of APCs. Their results showed that PCI caused a 30-fold increase in MHC class I/peptide complex formation and surface presentation on APCs, and a subsequent 30- to 100-fold more efficient activation of antigen-specific CTLs compared to using the peptide alone. These *in vitro* effects of PCI were translatable *in vivo* by the successful induction of antigen-specific CTL responses to cancer antigens in C57BL/6 mice following intradermal peptide vaccination and local light treatment. It is noteworthy that both macrophages and DC were used as APCs with approximately equal efficacy in these experiments. If these promising PCI strategies might be translatable to post-operative HGG treatment by the use of indwelling balloon light applicators, as has been proposed and tried for both radiation and PDT treatment, remains to be determined^[36-39]^.

### PDT for the treatment of brain tumors

PDT has been investigated as an adjuvant for the treatment of malignant gliomas for approximately 35 years^[39-43]^. Although numerous clinical trials have been initiated, the vast majority have consisted of uncontrolled phase I/II studies containing small numbers of patients. For example, in the single center phase III trial reported by Eljamel *et al*.^[39]^ using both flourecent guided resction combined with ALA and Photofrin repetitive PDT, a mean overall survival (OS) of the treatment group was 52.8 weeks compared to 24.6 weeks in the control group. In a phase II uncontrolled trial of 22 patients reported by Muragaki *et al*.^[41]^, using talaporfin sodium as a PS, a median of survival of 99 weeks was observed. This compared favorably to the 54-64 weeks OS obtained from previous trials employing standard post operative treatment consisting of radiation and TMZ. Due to differences in methodology and types of malignant brain tumors treated, it has been very difficult to evaluate PDT efficacy from these limited trials. For a more detailed account of the results of a number of PDT clinical trials for HGG, Bechet *et al*.^[42]^ and Quirk *et al*.^[43]^ give an excellent overview. Additionally, none of these clinical trials have included an evaluation of the effects of PDT on the immune response to treatment. Overall, the results of PDT trials for malignant gliomas have been relatively modest, thus providing the rationale for alternative PDT mediated treatment approaches such as PDT induced immunotherapy.

### PDT mediated immunity of brain tumors

There have been few experimental studies exploring the effects of direct PDT of brain tumors. Li *et al*.^[44]^ showed that PDT *in vivo* generated regional and systemic anti-tumor immunity in mice with G422 gliomas in the brain. The infiltration of immune cells and the release of inflammatory factors, such as TNF-α and IFN-γ, were increased in animals with G422 gliomas following PDT, compared to non-treated controls. Splenic lymphocytes, isolated from PDT-treated mice, were able to induce anti-tumor immunity in nude mice. These workers could also demonstrate that PDT induced anti-glioma immunity was significantly reduced in tumor bearing complement C3 knockout as well as in nude mice.

Although PDT/PCI has clearly demonstrated the induction of a significant anti-tumor immune response, light based therapies are limited by the rapid absorption of light in tissue. For this reason the therapeutic efficacy of PDT, using presently available PSs, has been clinically confined mainly to superficial relatively flat tumors limited to skin and head and neck surfaces as well as bladder and esophagus. Effective PDT has been shown to extend only up to a depth of approximately 4 mm in cerebral tissue^[45]^. It would therefore not affect the glioma cells in more distant infiltration zones in the resection cavity wall, which can be measured in cm. In addition, the tumor cells infiltrating normal brain that lead to tumor recurrence are protected by the blood brain barrier, so uptake of PS can be inadequate^[46]^. To overcome the difficulties of *in situ* light delivery and dosimetry in postoperative brain tumor resection cavities, PDT produced anti glioma vaccines are a related approach that takes advantage of the immune stimulatory effects of *ex vivo* PDT of tumor cell cultures.

## PDT-PRODUCED CANCER VACCINES FOR GLIOMA

### *Ex vivo* produced vaccines

In earlier studies, using extra cranial tumor models, Gollnick *et al*.^[47]^ demonstrated that lysates from PDT-treated tumor cells were more effective as preventative vaccines than tumor cells treated with UV, ionizing irradiation or cells subjected to freeze-thaw (F/T) cycles. Other groups have extended these results in several extra cranial models and could demonstrate that PDT-treated tumor cells could act as therapeutic anti-cancer vaccines^[48,49]^.

**Figure 2.**
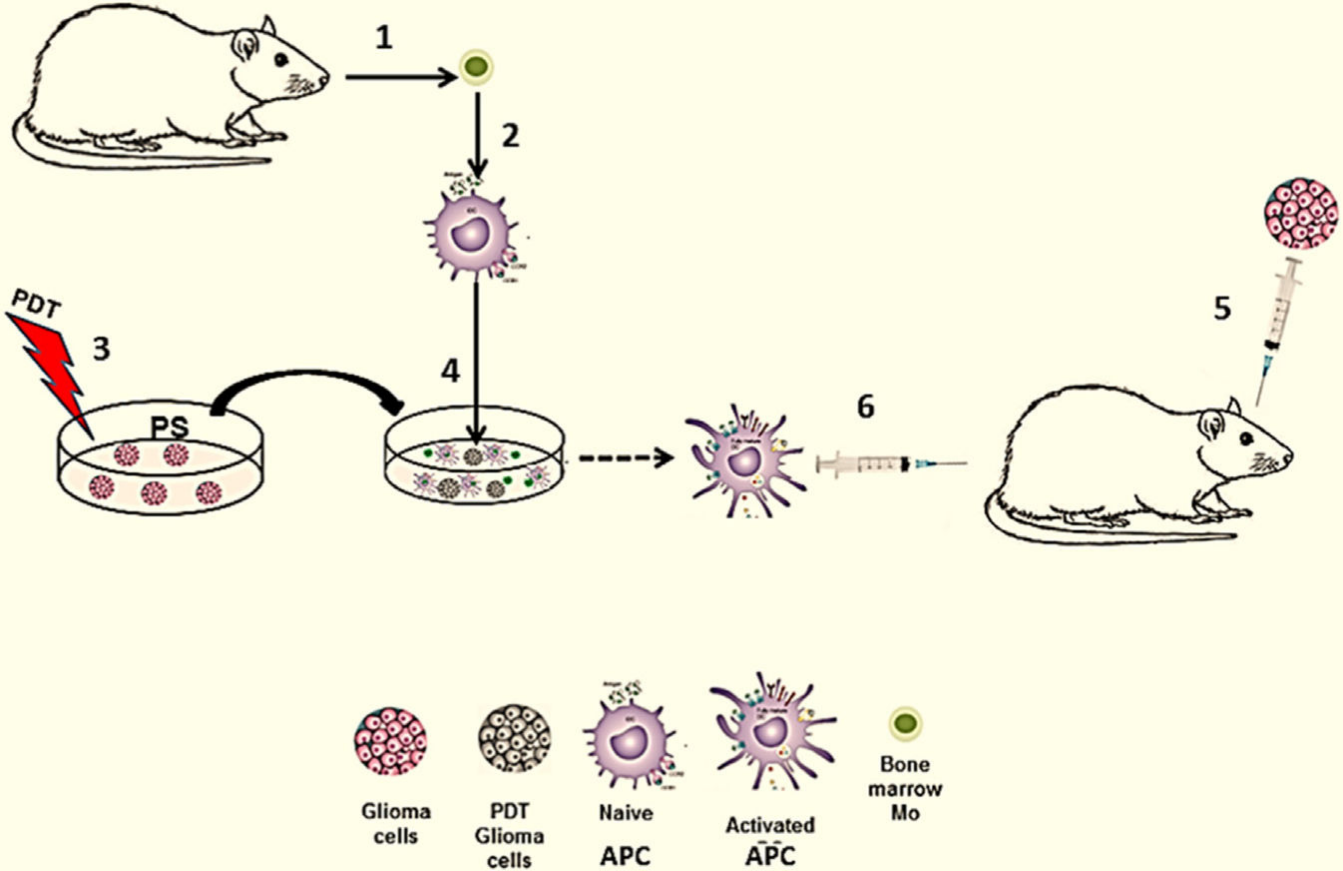
*Ex vivo* generated PDT-APC vaccine. (1) APC (DC/Ma) precursors obtained from donor animal; (2) cultured alone *in vitro* resulting in naïve APC; (3) *ex vivo* PDT treatment of tumor cells; (4) co-culture *in vitro* of treated tumor cells with naïve APC resulting in activated APC; (5) intra-cranial inoculation of glioma cells into the brain; (6) immunization with activated APC. APC: antigen presenting cell; PDT: photodynamic therapy; PS: photosensitizer

PDT generated vaccines against glioma cells have taken the form of CD activation by the use of crude tumor lysates, acid eluted crude lysates, and *in vitro* co-culture of PDT treated glioma cells and DC or macrophages (Ma) acting as APCs. Figure 2 illustrates the basic concept for an experimental PDT-APC co-culture vaccine.

The generation of vaccines in experimental models using PDT has been explored by a number of groups. For example, in an *in vitro* study, employing human glioma spheroids and dendritic cells from human donors, Etminan *et al*.^[50]^ (2011) showed that ALA-PDT of glioma spheroids *in vitro* promoted DC attraction, uptake of tumor antigens and maturation of DCs, three important initial steps of the afferent phase of adaptive immunity. Co-cultured DCs with ALA-PDT-treated spheroids promoted the induction of CD83 (a marker for mature DCs) and upregulation of the co-stimulatory molecules CD40, CD80 and CD86. Additionally HSP-70 was upregulated on the spheroids after ALA-PDT treatment.

Shixiang *et al*.^[51]^ generated DC vaccines using Photofrine-PDT-treated C6 glioma cell to produce antigenic peptides to activate DCs *ex vivo*. Immune response parameters between DC vaccines from PDT acid-eluted induced supernatants, DC vaccines from PDT-induced C6 supernatants or DCs exposed to antigens generated by direct acid elution only or freeze-thawing. Effects of these adaptively transfer DCs on host immunity were evaluated by measuring cytokine induction, as well as assessing DC-induced cytotoxic T lymphocyte (CTL)-mediated lysis of C6 target. Their results demonstrated that PDT-acid elution resulted in more effective DC differentiation associated with a high expression of CD80 and MHC-II compared with the other vaccine treatment groups. In addition the induction of the highest rat serum levels of IL-12 and TNFα and the lowest IL-10 levels were observed in the PDT acid eluted peptide group. Spleen cells isolated from these animals effectively mediated lysis of C6 target cells. They concluded that PDT of C6 cells significantly enhanced tumor cell immunogenicity compared to freeze-thawed C6 cells.

Reactive oxygen species (ROS) production and endoplasmic reticulum stress are believed to be important factors inducing ICD^[52]^. The photosensitizer Hypericin, a naturally occurring photosensitizer, mainly locates to the membranes of the endoplasmic reticulum and Golgi apparatus^[53]^. Hyp-PDT has been shown to induce major DAMPs characteristic of ICD including surface-exposed calreticulin (CRT), surface exposed HSP 70/90, secreted adenosine triphosphate (ATP), and passively released high-mobility group box 1 (HMGB1) protein^[54-56]^. ICD induced by Hyp-PDT was more effective in comparison to that induced by chemotherapy or radiotherapy^[57]^.

In a recent study Garg *et al*.^[58]^, combined HYP-PDT induced ICD with DC immunotherapy in an orthotopic HGG mouse model involving both prophylactic (immunization before i.c tumor cell implantation) as well as theraputic (immunization after i.c tumor cell implantation) treatment protocols. Both protocols using ICD-based DC vaccines demonstrated a significant anti-HGG survival benefit. In particular using a theraputic protocol, Hyp-PDT induced ICD-based DC vaccines together with chemotherapy (temozolomide) increased survival of HGG-bearing mice by up to 300%, resulting in half of the immunized animals becoming long-term survivors. Noteworthy was the observation that ALA-PDT treated glioma revealed no significant increase of CRT and release of HMGB1, two important DAMPs for the induction of ICD. The different characteristics of the various PSs used for PDT will in all probability determine their impact on subsequent antitumor immunity. Additionally, Hyp-PDT induced ICD-based DC vaccines appeared to induced an immune-stimulatory shift in the brain, from regulatory T cells to TH1/cytotoxic T lymphocyte/TH17 cells. A similar T cell shift has been shown to be associated with good patient prognosis in several tumor types^[59,60]^.

Although DCs have been used as APCs in the vast majority of immunization studies recent work has shown that DCs are part of the mononuclear phagocyte system and that they are indistinguishable from macrophages (Ma) in several key areas including developmental pathways, markers and efficacy as APCs^[61]^. Therefore, DCs it is argued, have no unique adaptation for antigen presentation that is not shared by other Ma and, as such, it is not surprising that both cell types are equally active vis a vis antigen presentation. We have used Ma together with the photosensitizer disulfonated aluminum phthalocyanine (AlPcS2a) mediated PDT of F98 rat glioma cells *ex vivo*. AlPcS2a is a photosensitizer which enters the cell by endocytosis, and tends to localize in endosomes and lysosomes. PDT at relatively low light dose rates causes partial damage to lysosomes resulting in the release of hydrolases, which trigger both apoptotic and/or autophagy cell death.

Fischer rats and F98 (syngeneic) and BT_4_C (allogeneic) glioma cells were used in these experiments, in an *in vivo* brain tumor development model^[62]^. Co-incubation of naive Ma with AlPcS2a-PDT treated F98 glioma cells led to pronounced morphological changes of the Ma. Naïve Ma were round in shape, approximately 10 to 15 μm in diameter and are composed of an equal population of both adherent and floating cells *in vitro*. By contrast, activated Ma were significantly larger, irregular in shape with increased intracellular inclusions and all of the cells were adherent in culture. Inoculation of these primed Ma (acting as APC), significantly inhibited but did not completely prevent the growth of F98-induced tumors in the brain. Complete suppression of tumor development though, was obtained via AlPcS2a-PDT-treated tumor cell primed Ma inoculation combined with direct intra-cranial injection of allogeneic glioma cells. Interestingly, allogeneic glioma cells injected into the brain in one hemisphere did not form tumors but surprisingly slowed the growth of syngeneic tumors induced in the contra-lateral hemisphere in the same animal. This appeared to indicate a systemic immune response generated via i.c inoculation by allogeneic glioma cells, though inadequate to prevent tumor development, did have an inhibiting effect.

Allogeneic cells likely contain antigen determinants shared with the syngeneic tumor, leading to the observed reduction in tumor growth. This hypothesis is in agreement with the previous findings of Stathopoulos *et al*.^[63,64]^ in preclinical studies in rats using both allo and syngeneic stimulation. The underlying DAMPs developed by AlPcS2a-PDT, as has been previouslydemonstrated for Hyp-PDT^[54-58]^, remains to be determined in detail.

### *In vivo* produced vaccines

**Figure 3.**
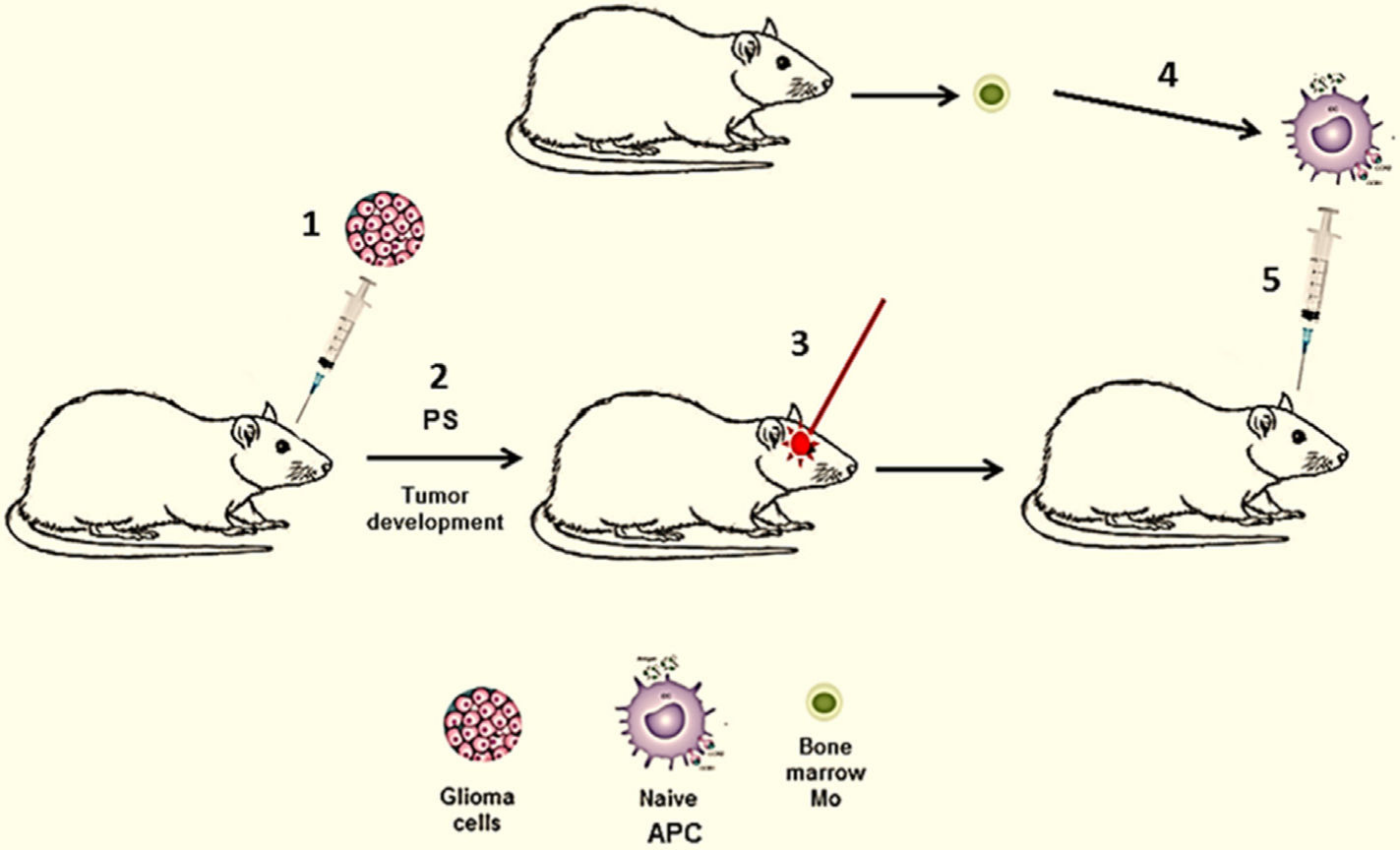
*In vivo* generated PDT-APC vaccine. (1) Intra-cranial inoculation of glioma cells; (2) tumor development, PS injection into the animal; (3) APC (DC/Ma) precursors obtained from donor animal, cultured alone *in vitro* resulting in naïve APC; (4) iPDT of tumor *in situ*; (5) immunization with naive APC injection directly into PDT treated tumor. APC: antigen presenting cell; PDT: photodynamic therapy; PS: photosensitizer

In all of the above mentioned studies glioma tumor cells were PDT treated *in vitro*. In a subset of brain tumor patients, harboring surgically inaccessible tumors, interstitial PDT (iPDT) has been evaluated^[65]^. Here light treatment is applied via stereotactically placed implantable fibers directly into the tumor. iPDT could be combined with direct injection of naïve APCs as has been done in several previously described extra-cranial experimental tumor models^[26-28]^. This protocol translated to intra-cranial tumors is illustrated in Figure 3 and is presently under development.

This combined iPDT-APC injection strategy would both directly destroy portions of the tumor and additionally induce *in situ* APC activation which enhances antigen acquisition and processing as well as migration of the APCs to draining lymph nodes. This *in vivo* produced vaccine would potentially enhance the primary PDT effect and prevent tumor regrowth. It would also obviate the time consuming and costly necessity of priming APCs *in vitro*.

## CONCLUSIONS

Although the experience with PDT/PCI produced anti HGG vaccines is limited and no clinical trials have been done, PDT/PCI appears to be an inducer of immunogenic cancer cell death, an important step in the afferent phase of the immune anti-tumor response. Light activated induced immunotherapy therefore holds the potential to become a complementary therapeutic option for for patients with HGG. Taking into account the penetration limitations of light activated therapies in the brain the further development of *ex vivo* PDT/PCI generated APC or peptide vaccines seems the most attractive approach. A deeper and detailed understanding of the induction of the antitumor immunity induced by light activated therapies would allow in the defining of protocols which would focus and enhance the immune system to recognize and prevent the inevitable post-operative recurrence of the tumor. Combining PDT induced anti-tumor vaccines with other therapeutic modalities including check-point inhibitors, is an exciting field to explore. Although not discussed in this review both PDT and PCI have an effect on the vasculature and have been show to temporarily open the blood brain barrier in a limited site specific region^[66-68]^. What additional role this would play in the development of an effective and safe anti-HGG patient therapy, remains to be established.

## Figures and Tables

**Table 1.** Typical photosensitizers and intracellular targets

Photosensitizer	Intra-cellular organelle	Cell death mechanism	References
5-aminolaevulinic acid (5-ALA)	Mitochondria (Mito)	Apoptosis	[8,46,50,65]
Hematoporphyrin (HMME)	Cell membrane (CM)	necrosis	[7,8,51]
Hypericin (HYP)	Endoplasmic reticulum (ER)	ICD	[8,20,54,58]
Disulfonated aluminum phthalocyanine (AIPcS2a)	Endosomes, Lysosomes (Lyso)	Apoptosis, autophagy ICD?	[8,15,29,31,62]

ICD: immunogenic cell death
